# Navigating the Future: A Comprehensive Review of Artificial Intelligence Applications in Gastrointestinal Cancer

**DOI:** 10.7759/cureus.54467

**Published:** 2024-02-19

**Authors:** Sreetama Mukherjee, Sunita Vagha, Pravin Gadkari

**Affiliations:** 1 Pathology, Jawaharlal Nehru Medical College, Datta Meghe Institute of Higher Education and Research, Wardha, IND

**Keywords:** collaborative research, diagnostic accuracy, personalized medicine, machine learning, artificial intelligence, gastrointestinal cancer

## Abstract

This comprehensive review explores the transformative role of artificial intelligence (AI) in the realm of gastrointestinal cancer. Gastrointestinal cancers present unique challenges, necessitating precise diagnostic tools and personalized treatment strategies. Leveraging AI, particularly machine learning and deep learning algorithms, has demonstrated remarkable potential in revolutionizing early detection, treatment planning, prognosis, and drug development. The analysis of current research and technological advancements underscores the capacity of AI to unravel intricate patterns within extensive datasets, providing actionable insights that enhance diagnostic accuracy and treatment efficacy. The transformative impact of AI on the landscape of gastrointestinal cancer is emphasized, signaling a paradigm shift towards more precise and targeted cancer care. The conclusion emphasizes the need for sustained research efforts and collaborative initiatives among AI researchers, healthcare professionals, and policymakers. By fostering interdisciplinary collaboration, we can navigate the evolving field of gastrointestinal cancer care, embracing the potential of AI to improve patient outcomes and contribute to a more effective and personalized approach to cancer management.

## Introduction and background

Gastrointestinal cancer poses a significant global health challenge, encompassing a spectrum of malignancies affecting various parts of the digestive system. From colorectal to pancreatic cancers, these diseases account for a substantial portion of cancer-related morbidity and mortality worldwide. As researchers and healthcare professionals strive to enhance diagnostic precision, treatment efficacy, and patient outcomes, the integration of artificial intelligence (AI) has emerged as a promising frontier in cancer research and therapy [1]. Gastrointestinal cancers, including those affecting the esophagus, stomach, liver, pancreas, and colorectal region, collectively represent a complex and diverse group of malignancies. These cancers often present unique diagnostic challenges due to their anatomical intricacies, diverse etiologies, and varied clinical manifestations. Understanding the nuances of gastrointestinal cancer is crucial for devising effective strategies for early detection, accurate diagnosis, and tailored treatment plans [2].

The convergence of AI and healthcare has brought forth transformative opportunities, particularly in the domain of cancer research and treatment. AI technologies, such as machine learning and deep learning algorithms, offer the potential to analyze vast datasets, identify subtle patterns, and derive meaningful insights from complex medical information. In the context of gastrointestinal cancer, the integration of AI holds the promise of revolutionizing early detection, optimizing treatment strategies, and advancing personalized medicine [3]. This comprehensive review aims to explore and evaluate the current landscape of AI applications in the field of gastrointestinal cancer. By examining the existing literature, case studies, and technological advancements, we seek to provide a thorough understanding of how AI is influencing and reshaping various aspects of gastrointestinal cancer research and treatment. The review will delve into AI-driven approaches in diagnosis, treatment planning, prognosis, drug development, and research, shedding light on the challenges, ethical considerations, and future directions in this dynamic intersection of technology and healthcare.

## Review

Gastrointestinal cancer: an overview

Classification of Gastrointestinal Cancers

The categorization of gastrointestinal (GI) cancers encompasses a range of types, including esophageal, gastric, colorectal, pancreatic, liver, and anal cancers, among others. These cancers exhibit diversity in terms of etiology and clinical management, each type associated with distinct risk factors and symptoms. For instance, gastric cancers can be broadly categorized into two primary topographical subsites: the cardia and non-cardia, differing in etiology, risk factors, and geographical patterns [1]. Esophageal cancer, with approximately 572,000 new cases and 508,000 deaths in 2018, ranks as the seventh most prevalent cancer globally. Conversely, colorectal cancer stands out as one of the most common and highly treatable GI cancers in the United States [1,4]. The American Cancer Society advocates for routine colorectal cancer screening, emphasizing the critical importance of early detection for effective treatment. Gastrointestinal cancers demonstrate a higher propensity to develop in men, with the risk escalating with age. Symptoms manifest differently based on the type of cancer, ranging from difficulty swallowing in esophageal cancer to ulcer-like symptoms in gastric cancer [4].

Incidence and Prevalence Statistics

The occurrence and frequency of gastrointestinal cancers, encompassing gastric and colorectal cancers, exhibit variations influenced by region, gender, and the Human Development Index (HDI). Over the period of 2015-2019, the average annual incidence rate was 33% higher in men (41.5 per 100,000) compared to women (31.2 per 100,000) [5]. Global incidence rates demonstrated an upward trajectory corresponding to increased HDI levels, ranging from 104.3 to 128.0 per 100,000 [2]. Gastric cancer, more frequently diagnosed in males, accounts for 66% of all cases in men, with incidence rates varying from less than five to 20 per 100,000 across diverse HDI nations [6,7]. The incidence of gastric cancer displays notable regional and cultural variability, with Eastern and Central Asia and Latin America reporting the highest rates. Globally, it ranks as the third leading cause of cancer-related deaths, contributing to approximately 1 in 12 oncological fatalities [7]. In 2020 alone, an estimated 1.1 million new cases and 770,000 deaths were attributed to gastric cancer, with incidence rates being, on average, twice as high in males compared to females (15.8 and 7.0 per 100,000, respectively) [6]. These statistics underscore significant gender and regional disparities in the occurrence and prevalence of gastrointestinal cancers, emphasizing the imperative for targeted screening, prevention, and treatment strategies.

Current Challenges in Diagnosis and Treatment

The diagnosis and treatment of gastrointestinal (GI) cancers, particularly gastric cancer, confront several significant challenges. Among these challenges are notable delays in both diagnosis and treatment, the intricate genetic complexity and heterogeneity inherent in gastric cancer, and the imperative for standardization in diagnosis to identify eligibility for emerging therapies. A research study conducted in Nepal sought to quantify and pinpoint the causes of delays in the diagnosis and treatment of GI cancer patients, shedding light on the formidable barriers posed by time delays in the management of these cancers [8]. Moreover, the genetic intricacies and heterogeneity associated with gastric cancer have posed substantial obstacles to advancements in treatment [9].

AI has emerged as a progressively applied solution in response to these challenges. AI technologies have been employed to forecast tumor recurrence, formulate tailored treatment plans, and standardize the diagnostic process in gastric cancer. These applications are designed to enhance patient outcomes and furnish valuable guidance for physicians in their decision-making endeavors [10,11]. Additionally, AI tools have been developed to predict both overall survival and disease-free survival subsequent to colorectal cancer diagnosis, offering potential advantages in clinical decision-making [12]. These findings underscore the considerable potential of AI in mitigating existing challenges in the diagnosis and treatment of GI cancers, including gastric cancer, by furnishing more precise and timely insights for patient management.

AI in cancer diagnosis

Role of AI in Early Detection

Image-based diagnostics: AI plays a pivotal role in advancing early detection methodologies, mainly through image-based diagnostics in the field of cancer. AI-based diagnostic tools have demonstrated a capacity to augment the accuracy and speed with which complex medical images are interpreted, consequently enhancing the early detection of diseases, including cancer. Leveraging machine learning algorithms, AI systems exhibit the capability to swiftly and precisely analyze medical images, facilitating the identification of early-stage diseases that might pose challenges for traditional detection methods [13]. Notably, AI-enabled medical imaging tools contribute to the automation of image analysis, diminishing manual workloads and potentially expediting diagnoses [14]. Moreover, AI has showcased remarkable accuracy and sensitivity in identifying imaging abnormalities, holding promise for refining tissue-based detection and improving overall disease detection outcomes [15]. In the realm of cancer, AI-enabled imaging and diagnostics present significant potential for elevating disease detection standards and aiding clinicians in diagnosing and characterizing cancer aggressiveness. For instance, ongoing AI research endeavors aim to enhance disease detection capabilities, such as identifying adenomas in colorectal cancer and evaluating the severity of prostate cancer [16]. In essence, the integration of AI into image-based diagnostics holds immense promise for advancing early detection and diagnosis of cancer, ultimately contributing to superior patient outcomes.

Radiology and medical imaging: Radiology and medical imaging represent interconnected fields within medical diagnostics. Radiology, a medical branch utilizing radiant energy for disease diagnosis and treatment, intertwines with medical imaging-a technology employed by specialized medical practitioners known as radiologists, particularly for diagnostic purposes [17,18]. Radiologists, medical doctors specializing in interpreting diagnostic images, employ various imaging technologies, such as x-rays, computed tomography (CT) scans, magnetic resonance imaging (MRI), mammography, nuclear medicine, and ultrasound, to diagnose and treat patients [19,20]. These medical imaging technologies hold the potential to enhance early detection and aid clinicians in diagnosing and assessing the aggressiveness of diseases, including cancer [18]. Interventional radiologists utilize imaging technologies like CT, ultrasound, MRI, and fluoroscopy to assist in the diagnosis and treatment of patients [18,19]. In summary, radiology and medical imaging play a pivotal role in diagnosing and treating various diseases, including cancer, with the potential to significantly enhance patient outcomes.

Pathology and histopathology: The field of pathology and histopathology has undergone substantial transformation through the integration of AI, particularly in the realm of cancer diagnosis. AI applications in pathology, also termed computational pathology, have been developed to analyze histopathology images, resulting in more efficient and accurate diagnoses. These AI tools aim to save pathologists time, minimize errors, and provide more objective and consistent pathology reports [21,22]. In diagnostic pathology, AI technology has the potential to automate time-consuming tasks, enabling pathologists to deliver fast and reliable diagnoses and focus on high-level decision-making. Additionally, AI can support the overall reporting system, expediting reporting time and measuring morpho-biological features more objectively [22]. The use of AI in pathology is anticipated to alleviate the workload for human experts, enhance the objectivity and consistency of pathology reports, and have a clinical impact by extracting hidden information from routinely available data [23]. Furthermore, AI has the potential to predict cancer outcomes, treatment responses, genetic alterations, and gene expression from digitized histopathology slides, thereby advancing cancer research and clinical oncology [23].

Biomarker identification and validation: Crucial to developing effective diagnostic and therapeutic strategies for cancer, biomarker identification and validation have witnessed increased utilization of AI. AI contributes significantly to biomarker discovery and validation by integrating various data types, including genomics, proteomics, and imaging data. Following the identification of candidate biomarkers, two validation methods-analytical and clinical-are employed. Analytical validation assesses the performance characteristics of a biomarker, such as sensitivity, specificity, accuracy, precision, and interlaboratory reproducibility, adhering to a predetermined protocol. Clinical validation seeks to establish a meaningful association between a biomarker and the disease, determining the biomarker’s utility and usefulness in a clinical context. This validation process often involves external validation through retrospective analysis of clinical trial data or prospective clinical trials [24]. AI and machine learning algorithms are instrumental in integrating diverse data types, expediting the process of biomarker discovery and validation, streamlining drug development, and ultimately enhancing patient outcomes [25-27].

Machine Learning Algorithms for Risk Assessment

AI and machine learning algorithms have demonstrated significant promise in the realm of early disease detection, particularly in the context of cancer. These advanced algorithms can analyze vast sets of medical data to identify risks and predict the potential spread of diseases based on symptoms and genetic history [28]. AI-based diagnostic tools play a crucial role in accelerating the interpretation of intricate medical images, leading to enhanced early detection of diseases and ultimately contributing to improved outcomes for patients [13]. For example, AI has proven effective in detecting mammographic abnormalities, showcasing a comparable accuracy to that of radiologists in breast cancer imaging [29]. Furthermore, AI applications have been successfully employed in identifying the early stages of lung cancer and colon cancer with high accuracy [30]. Notably, in the context of breast cancer, AI has contributed to a noteworthy improvement in early detection rates [30]. The transformative potential of AI lies in its ability to revolutionize early cancer diagnosis by recognizing patterns and anomalies that may signify the presence of disease or injury. This capacity enables the earlier detection of diseases, ultimately leading to improved patient outcomes [30]. The integration of AI in early disease detection signifies a paradigm shift in healthcare, offering a more efficient and precise approach that holds great promise for the advancement of patient care.

Comparison of Traditional Methods With AI-Enhanced Diagnostic Approaches

A comparison between traditional methods of cancer diagnosis and AI-based approaches, as emphasized in recent literature, reveals distinctive characteristics in various aspects. AI exhibits the potential to detect cancer at earlier stages by efficiently analyzing extensive imaging data, identifying subtle changes in scans, and managing large volumes of data, thereby making mass screenings more feasible. Furthermore, AI holds promise in assisting the characterization of tumors at an early stage, contributing to the identification of their nature and behavior. In contrast, traditional methods may necessitate more time for image analysis or laboratory tests, potentially leading to delays in diagnosis. While traditional methods have historically served as the cornerstone of cancer diagnosis and are considered reliable, they often entail significant costs, involve substantial infrastructure and machinery, and may lack precision in diagnosis. An illustrative example of AI's superiority is evident in its outperformance of existing clinical tests in identifying the risk of cancer in nodules [31]. AI models have demonstrated the capability to automatically detect and diagnose gastrointestinal tumors with comparable accuracy to expert clinicians, showcasing significant potential in enhancing diagnostic precision and facilitating early detection. For instance, the Gastrointestinal AI Diagnostic System (GRAIDS) achieved an impressive diagnostic accuracy of 95.5% in identifying upper gastrointestinal cancers, underscoring its potential as a real-time AI system for early gastric cancer diagnosis [32]. These advancements highlight the transformative impact of AI in revolutionizing cancer diagnosis, presenting a more efficient, cost-effective, and precise alternative to traditional methods.

AI in treatment planning and personalized medicine

Optimization of Treatment Strategies Through AI

AI is increasingly becoming a cornerstone in the optimization of treatment strategies, particularly within the realm of radiotherapy. AI-based treatment planning employs diverse approaches, including automated rule implementation, reasoning, modeling of prior knowledge, and multicriteria optimization. These methods aim to automate the planning process, optimize dosimetric trade-offs, and enhance the efficiency of medical science. For example, AI has been instrumental in developing a dose-volume histogram (DVH)-guided intensity-modulated radiation therapy (IMRT) optimization algorithm for automatic treatment planning and adaptive radiotherapy replanning. Furthermore, AI can revolutionize radiation therapy by reducing the time required for treatment planning, advocating knowledge-based treatment planning, and refining plan quality through the iterative improvement of AI models. The incorporation of AI into treatment planning holds significant promise for elevating the planning process's accuracy, efficiency, and robustness [33-35].

Additionally, AI is pivotal in advancing personalized medicine by identifying appropriate intervention targets and tailoring treatment strategies for individual patients. By analyzing vast datasets, AI contributes to creating tailored treatment plans and predicts patient responses to specific interventions, ultimately leading to enhanced patient outcomes. The application of AI in the development of personalized medicine focuses on identifying underlying pathologies, determining suitable interventions, and evaluating their efficacy, thereby revolutionizing the treatment landscape for various conditions [36]. Integrating AI in treatment optimization and personalized medicine signifies a paradigm shift in healthcare, offering a more precise, efficient, and patient-centric approach to medical intervention.

Integration of Genomics and AI for Personalized Treatment Plans

Genomics and AI convergence hold tremendous potential for developing personalized treatment plans. Through integrative strategies and multidisciplinary knowledge, AI is pivotal in formulating personalized treatment plans by discerning patterns in gene sequences or molecular signatures. This capability enables the identification of treatments that are most likely to be effective, thereby facilitating personalized diagnosis and prognostication [37]. The effectiveness of AI in advancing personalized medicine is contingent on its adeptness at aggregating, accessing, and integrating data derived from genomics and other sources. This process leads to the creation of tailored and optimized treatment strategies uniquely suited to individual patients [36]. Integrating AI and genomics can transform healthcare by replacing the conventional one-size-fits-all approach with more precise and effective therapies, ultimately resulting in improved patient outcomes [38]. This paradigm shift underscores the transformative impact of AI in shaping the future of personalized medicine, offering a more targeted and sophisticated approach to treatment planning and patient care.

Case Studies Demonstrating Successful AI-Driven Treatment Interventions

Numerous case studies provide compelling evidence of successful AI-driven treatment interventions. One illustrative example involves an AI model trained on an extensive dataset incorporating patient records, genetic information, and treatment outcomes. This model has proven effective in generating potential treatment plans tailored to individual patients, ultimately improving patient outcomes [39]. Another noteworthy application of AI involves enhancing ongoing psychological interventions for real-time emotional issues, leading to enhanced treatment outcomes [40]. Additionally, AI has been instrumental in the development of efficient tools for personalized medicine, including predictive models that forecast illness, treatment responses, and preventive measures [36,41]. While these case studies underscore the considerable potential of AI in healthcare, further research is imperative to comprehensively understand the broader impact of AI-driven treatment interventions on patient outcomes. Continued investigation will provide valuable insights into the efficacy and long-term implications of integrating AI into treatment strategies across various medical domains.

AI in prognosis and predictive analytics

Predictive Modeling for Patient Outcomes

Predictive modeling in healthcare, facilitated by AI, presents numerous benefits and applications, employing mathematical or computational methods to create models that forecast future outcomes. In the context of patient care, predictive modeling contributes to improved care outcomes, decreased readmissions, and heightened patient satisfaction rates by enabling hospitals to provide proactive and personalized care. It facilitates the identification of high-risk patients and their vulnerability to diseases, thereby enhancing patient management and disease prevention [42].

Predictive modeling has applications in various clinical areas within medicine, including cardiology, cancer characterization, and surgery outcomes. For instance, in cancer care, predictive modeling has been utilized to create models predicting the survival of patients with specific types of cancer, such as cervical cancer. These models leverage patient-specific clinical, physical, and dosimetric parameters to enhance prognostic accuracy [43].

While predictive modeling holds significant promise for identifying patients at the highest risk for adverse events and improving overall patient outcomes, it is imperative to ensure the transparency and explainability of these models. Explainable AI (XAI) becomes crucial in gaining the trust of clinicians and understanding the application of predictive modeling in healthcare. XAI addresses the need for transparency, monitors model performance, handles missing or inconsistent data, and enhances model performance across clinical scenarios or patient populations [44]. This emphasis on explainability ensures that predictive modeling delivers accurate predictions and fosters understanding and trust among healthcare professionals, ultimately optimizing its integration into clinical practice.

Identification of Prognostic Factors Using AI

AI is increasingly becoming a valuable tool for identifying and validating prognostic factors in diverse medical conditions, with a significant focus on applications in cancer. In gastrointestinal cancer, AI algorithms, such as the minor absolute shrinkage and selection operator (LASSO), have been successfully employed to identify and validate prognostic factors in patients [45]. Furthermore, AI methods have been applied to develop prognostic models specifically for surgically resected non-small cell lung cancer, demonstrating the potential of AI in conducting robust prognostic analyses [46]. AI is also making strides in exploring patient datasets to forecast the likelihood of various medical conditions, including rare hereditary and neurodegenerative diseases, thereby contributing valuable insights to prognostic analysis and personalized medicine [47]. Within this context, machine learning models have been instrumental in identifying prognostic and predictive cancer biomarkers, underscoring the potential of AI in advancing prognostic analysis and personalized medicine [48]. These findings underscore the expanding role of AI in identifying prognostic factors and developing prognostic models across a spectrum of medical conditions, prominently in the field of cancer. The integration of AI in prognostic analysis holds promise for enhancing patient care and health outcomes by providing more accurate prognostic assessments and formulating personalized treatment strategies tailored to individual patient needs.

Applications of AI in Predicting Treatment Response

Antidepressant response prediction: AI-assisted machine learning models have been developed to forecast the response to various antidepressant classes using electronic health records (EHR) data. Demonstrating promising results, these models have achieved area under the receiver operating characteristic curve (AUROC) values of at least 0.70 and area under the precision-recall curve (AUPRC) values of 0.68 or higher [49].

Immunotherapy response prediction: AI models have undergone validation to anticipate patients' responses to immunotherapies before the commencement of treatment. This capability aids in identifying patients likely to benefit from immunotherapy and those less likely to respond, ultimately enhancing patient outcomes [50].

Chemotherapy resistance prediction: AI and machine learning techniques have been applied to predict the likelihood of cancer developing resistance to chemotherapy. In cervical cancer, for instance, AI accurately identified tumors susceptible to therapy, improving patient outcomes. Additionally, the model pinpointed tumors likely to resist treatment, offering valuable insights for formulating personalized treatment strategies [51].

Drug interaction prediction: AI algorithms can analyze extensive datasets of patient information to identify potential drug interactions. This functionality aids in reducing the risk of adverse drug reactions, improving patient outcomes, and cutting healthcare costs [52].

Population health management: AI-driven predictive analytics enhance the accuracy, efficiency, and cost-effectiveness of disease diagnosis and clinical laboratory testing in population health management. This, in turn, improves patient outcomes, reduces healthcare costs, and enhances the precision and efficiency of drug dosing [52]. As AI continues to evolve, its increasing role in predicting treatment responses and supporting clinical decision-making is anticipated. However, addressing potential challenges and ensuring these technologies' responsible and equitable integration into healthcare practice remains essential.

Case studies and success stories

Highlighting Real-World Examples of AI Applications in Gastrointestinal Cancer

AI presents a transformative potential in various gastrointestinal (GI) cancer management aspects, as exemplified in real-world applications. In endoscopic examinations, AI is a valuable tool for enhancing early detection. It aids in identifying precancerous lesions and early tumors while also providing estimates of tumor invasion depth and precise delineation of resection margins [53]. In pathological diagnosis, AI has proven its capabilities by achieving an impressive area under the curve (AUC) of 0.92 in accurately diagnosing gastric cancer. This performance surpasses human experts, highlighting AI's precision and efficiency in the diagnostic process [53,54].

AI's proficiency extends to predicting submucosal invasion in early gastric cancer, where it achieves an AUC of 0.86, outperforming human experts in delivering accurate assessments [55]. In the context of preoperative staging for gastric cancer, AI predicts the depth of tumor invasion with an AUC of 0.88, once again surpassing the capabilities of human experts and providing crucial insights for informed preoperative decisions [55]. The application of AI in predicting the recurrence of gastric cancer after surgery is marked by notable success, with an AUC of 0.81. This surpasses the performance of human experts and offers valuable insights for post-surgical management and surveillance [55].

Moreover, AI leaps personalized treatment decision support for gastric cancer patients, showcasing its ability to outperform human experts in generating tailored and optimized treatment plans. These examples underscore the significant impact of AI in enhancing early detection and accurate diagnosis and providing personalized treatment strategies that contribute to optimal patient outcomes [55]. As AI technologies continue to advance, their role in the comprehensive management of GI cancers is expected to grow, promising improvements in early detection, precise diagnostics, and the formulation of personalized treatment strategies.

Positive Outcomes and Impact on Patient Care

Improved diagnostic speed and accuracy: AI algorithms substantially advance healthcare by swiftly and accurately processing large datasets. In diagnostics, AI can analyze medical images such as X-rays and MRI scans, identifying patterns and anomalies that human providers may overlook. This capability leads to faster and more precise diagnoses, ultimately improving patient outcomes [56].

Personalized treatment decision support: The application of AI extends to developing personalized treatment plans for gastric cancer patients. AI algorithms continuously monitor patients' health data, offering recommendations for lifestyle changes and treatment strategies tailored to individual needs. This personalized approach contributes to better patient outcomes and an enhanced quality of life [56].

Reduced healthcare costs: AI provides real-time data and recommendations, aiding healthcare providers in proactive patient management. By monitoring vital signs like blood pressure and heart rate, algorithms can identify potential health issues before they escalate. This preventive approach helps mitigate serious health complications, reducing healthcare costs [56].

Improved access to care: AI algorithms enhance healthcare accessibility, especially in remote and underserved areas. Telemedicine platforms powered by AI facilitate medical consultations and treatment recommendations for patients in the comfort of their homes, extending care to a broader population [56].

Enhanced patient engagement: AI empowers patients to manage their health actively. Fueled by AI, wearable devices monitor physical activity, sleep patterns, and other health metrics, enabling patients to make informed lifestyle changes. This proactive engagement contributes to improved health and a reduced risk of developing chronic conditions [56].

Improved patient safety outcomes: AI is crucial in identifying health risks, significantly impacting patient safety outcomes. Studies demonstrate high accuracy in AI as a diagnostic and prognostic tool, aiding in classifying patients based on ailments and severity. It helps identify common incidents, such as fall risks, delivery delays, and adverse drug effects, thereby minimizing risks to patient safety [57].

Streamlined diagnoses and improved clinical outcomes: AI contributes to healthcare efficiency by automating tasks and analyzing extensive patient datasets. This data-driven approach provides clinical decision support to healthcare professionals, streamlining diagnoses and leading to improved clinical outcomes [58].

## Conclusions

In conclusion, the integration of AI in gastrointestinal cancer holds immense promise, as evidenced by the essential findings and insights derived from this comprehensive review. The application of AI technologies, including machine learning and deep learning algorithms, has showcased transformative potential across various facets of cancer care. From bolstering early detection capabilities to optimizing personalized treatment plans, AI stands as a beacon of innovation in addressing the intricacies of gastrointestinal malignancies. Its ability to analyze extensive datasets, discern subtle patterns, and provide actionable insights signifies a paradigm shift that can significantly enhance diagnostic accuracy and treatment efficacy. Emphasizing AI's transformative impact on gastrointestinal cancer's landscape, this review underscores the need for continued research and collaborative efforts. By encouraging interdisciplinary partnerships among AI researchers, healthcare professionals, and policymakers, we can foster a dynamic environment conducive to ongoing refinement and innovation. As we navigate the future of gastrointestinal cancer care, the commitment to exploring emerging technologies and sharing knowledge remains crucial for improving patient outcomes and shaping a more effective, personalized approach to cancer management.
